# Efficacy of Disitamab Vedotin and Immune Profiles in High‐Risk Non‐Muscle‐Invasive Bladder Cancer With HER2 Overexpression

**DOI:** 10.1002/mco2.70427

**Published:** 2025-10-07

**Authors:** Haoyang Liu, Junru Chen, Qiyu Zhu, Haolin Liu, Yeechun Chuang, Mengni Zhang, Guangxi Sun, Hao Zeng

**Affiliations:** ^1^ Department of Urology Institute of Urology, Sichuan Clinical Research Center for Kidney and Urologic Diseases, West China Hospital, Sichuan University Chengdu China; ^2^ Department of Pathology West China Hospital, Sichuan University Chengdu China

1

Dear Editor

Following transurethral resection of bladder tumor (TURBT), intravesical Bacille Calmette‐Guerin (BCG) instillation remains the gold‐standard adjuvant immunotherapy for high‐risk non‐muscle‐invasive bladder cancer (HR‐NMIBC). However, 5‐year recurrence and progression rates still reach 58% and 35%, respectively, among HR‐NMIBC patients receiving BCG [1]. Human epidermal growth factor receptor 2 (HER2) overexpression has been identified as an independent predictor of poor response to BCG in NMIBC. Patients with HR‐NMIBC and HER2 overexpression exhibited a higher risk of disease recurrence and progression following BCG treatment [1]. Disitamab vedotin (DV; RC48‐ADC) is a HER2‐targeted antibody–drug conjugate (ADC) comprising a humanized HER2‐directed antibody conjugated to monomethyl auristatin E via a cleavable linker [2]. Clinical trials have demonstrated promising efficacy and manageable safety of DV in metastatic urothelial carcinoma patients with HER2 overexpression [3]. This cohort study aims to compare the efficacy of DV and BCG in HR‐NMIBC patients with immunohistochemistry (IHC)‐confirmed HER2 overexpression and evaluate the relationship between HER2 expression and tumor immune profiles.

From March 2022 to February 2024, 11 HR‐NMIBC patients (median age: 66 years; 3 females) with HER2 overexpression received DV (120 mg intravenously every 2 weeks; median treatment cycle: six cycles; range: six to eight cycles) after TURBT and second‐confirmed TURBT at West China Hospital of Sichuan University. Seventeen patients (median age: 63 years; three females) receiving adjuvant BCG instillation (median treatment cycle: 19 cycles; range: 6–27 cycles) were matched by clinicopathologic features (details are available in Supporting Information Methods). The median follow‐up durations were 19.2 months for the DV cohort and 37.0 months for the BCG cohort. Kaplan–Meier analysis indicated that patients receiving DV exhibited comparable RFS to those receiving BCG (Figure 1A, *p* = 0.068). Notably, the 12‐month RFS rate was 100% for the DV group, compared with 58.8% for the BCG group.

**FIGURE 1:**
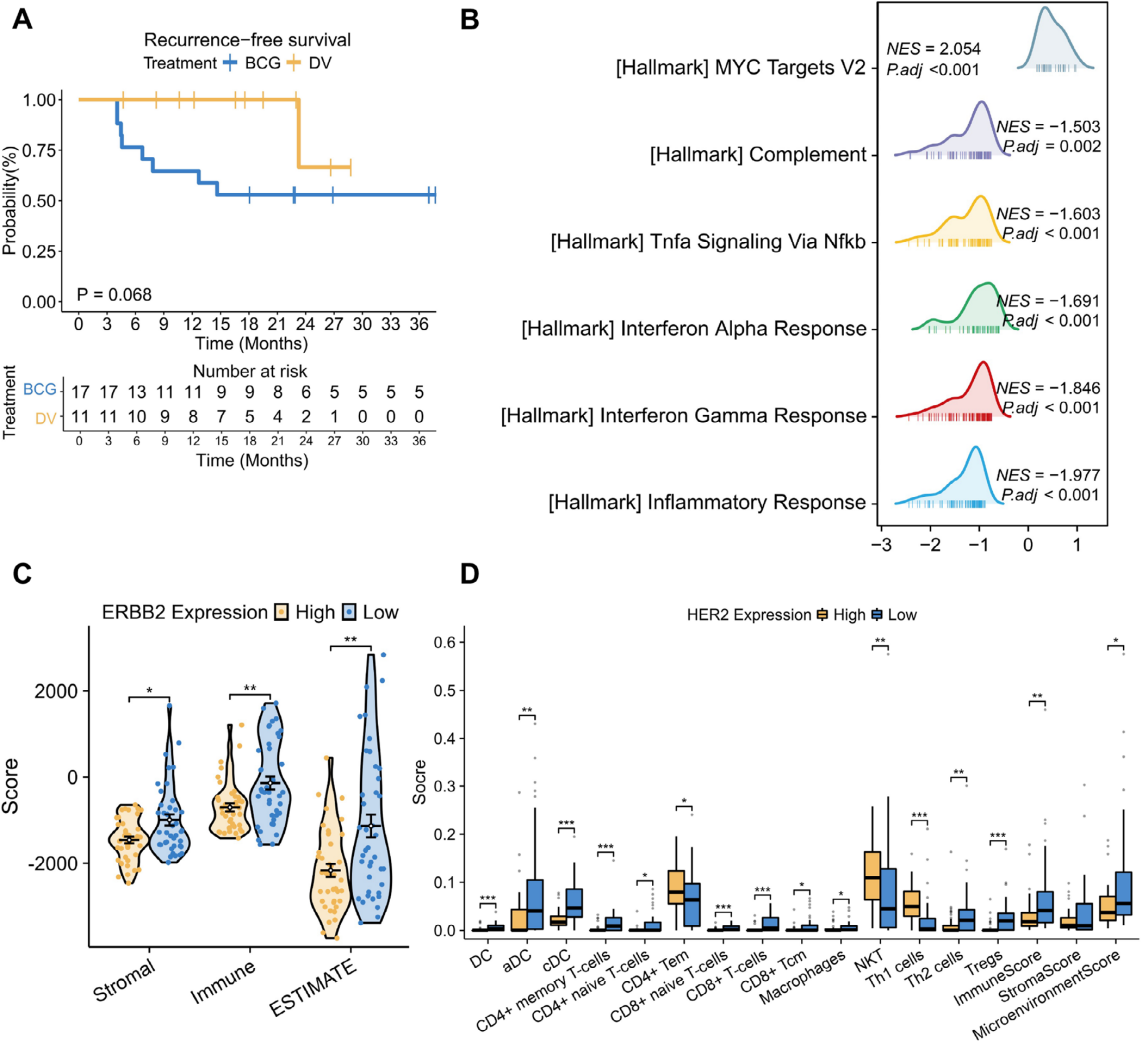
Survival outcomes of BCG‐treated and DV‐treated patients and transcriptional characteristics of high‐risk non‐muscle‐invasive bladder cancer (HR‐NMIBC) stratified by *ERBB2* expression. (A) Kaplan–Meier curve comparing recurrence‐free survival in HR‐NMIBC patients with HER2 overexpression who received BCG (*n* = 17) versus DV (*n* = 11) as adjuvant treatment. (B) Enrichment of immune‐related hallmark pathways in BCG‐naive HR‐NMIBC tumors with high *ERBB2* expression compared to tumors with low *ERBB2* expression, identified via gene set enrichment analysis. (C) Bean and box plots illustrating correlations between *ERBB2* expression and immune score, stromal score, and ESTIMATE score in BCG‐naive HR‐NMIBC, analyzed using the ESTIMATE algorithm. The means ± SEM for data from the *ERBB2* high (*n* = 40) and low (*n* = 40) groups are represented by the error bars. (D) Box plots showing differences in immune infiltration between the *ERBB2* high‐expression and low‐expression groups in BCG‐naive HR‐NMIBC analyzed using the xCell algorithm. The means ± SEM for data from the *ERBB2* high (*n* = 40) and low (*n* = 40) groups are represented by the error bars. adj, adjust; aDC, activated dendritic cell; BCG, Bacille Calmette‐Guerin; cDC, conventional DC; DC, dendritic cell; DV, disitamab vedotin; NES, normalized enrichment score; NKT, natural killer T cell; SEM, standard error of the mean; Tem, effector memory T cell; Th, T helper; Treg, regulatory T cell. *p < 0.05; **p < 0.01; ***p < 0.001.

The most common treatment‐related adverse events (TRAEs) were alopecia (63.6%) and bladder irritation (70.6%) for the DV group and BCG group, respectively. Other common TRAEs in the DV group included asthenia (54.5%), decreased appetite (54.5%), nausea (54.5%), blood triglycerides increased (54.5%), blood glucose increased (54.5%), pruritus (36.4%) and pyrexia (36.4%). In the BCG group, common TRAEs included fever (47.1%), fatigue (29.4%), and macroscopic hematuria (17.6%). Overall, DV demonstrated promising efficacy and manageable toxicity, suggesting its potential as an alternative treatment for patients with HR‐NMIBC.

To further investigate the impact of HER2 overexpression on immune profiles of NMIBC, we analyzed transcriptome data from 132 primary tumors of BCG‐naive HR‐NMIBC patients [4], as reported by de Jong et al. Based on *ERBB2* mRNA expression, 80 patients were selected and divided into *ERBB2*‐high (the highest 40 cases, 30%) and *ERBB2*‐low (the lowest 40 cases, 30%) expression groups. Gene set enrichment analysis (GSEA) based on hallmark pathways revealed that the *ERBB2*‐high group was significantly associated with lower expression of immune‐related pathways, including the complement, TNF‐α, interferon alpha and gamma responses, and inflammatory response pathways (Figure 1B). GSEA analyses based on KEGG and GO pathways also showed downregulated pathways involving immune response and immune cells, including natural killer cells and T cells, in *ERBB2*‐high group. Immune microenvironment analyses via ESTIMATE and XCell algorithms demonstrated significantly lower stromal, immune, and estimate scores in the *ERBB2*‐high group compared to the *ERBB2*‐low group (Figure 1C). Moreover, CD4^+^ and CD8^+^ T cells, along with macrophages and dendritic cells that positively regulate T‐cell activation, were less infiltrated in *ERBB2*‐high tumors (Figure 1D). In summary, HR‐NMIBC tumors characterized by high *ERBB2*/HER2 expression display a markedly suppressed immune microenvironment, indicating that patients within this subgroup may derive limited benefit from immunotherapy.

The incidence of HER2 overexpression (IHC ≥ 2+) in HR‐NMIBC reached 41%, and among these patients, the BCG efficacy significantly diminished, with 5‐year RFS and PFS rates of 19% and 40%, respectively [1]. To date, no studies have reported on the efficacy of HER2‐targeted ADC, such as DV, in HR‐NMIBC. This study provides initial evidence that DV achieves comparable RFS to BCG, offering a potential alternative adjuvant therapy for HR‐NMIBC patients with HER2 overexpression.

Moreover, we investigated potential mechanisms attributed to poor response to BCG in HER2‐overexpressed NMIBC. We found that NMIBC tumors with HER2 overexpression exhibited a distinct immune profile characterized by downregulated immune pathways and reduced infiltration of T cells and immune‐regulating cells—factors that have been reported to affect BCG efficacy [5]. In this context, DV may serve as an alternative treatment option, not only alleviating the global BCG shortage but also benefiting HER2‐positive HR‐NMIBC cases with poor response to BCG. To validate these findings, we have initiated a phase II clinical trial (NCT05996952) to further investigate the efficacy and safety of DV in this specific patient population.

## Author Contributions

H.Y.L, J.R.C., and Q.Y.Z. contributed equally to this work. G.X.S. and H.Z. designed the study. H.Y.L. and J.R.C. wrote the manuscript. H.Y.L., J.R.C., Q.Y.Z., Y.C.C., and M.N.Z. were involved in the acquisition, analysis, and interpretation of data. H.Y.L. and J.R.C. performed statistical analyses. H.Y.L., Q.Y.Z., and H.L.L. designed the figures. G.X.S. and H.Z. provided critical revision of the manuscript. All authors read and approved the final manuscript.

## Ethics Statement

This study was conducted in accordance with the Declaration of Helsinki was approved by the Ethics Committee of West China Hospital of Sichuan University. Written informed consent was obtained from all participants, and the ethical approval number was 2020(1009).

## Conflicts of Interest

The authors declare no conflicts of interest.

## Supporting information




**Supplementary Methods and Materials: Supplementary Table 1**: Clinicopathological characteristics in high‐risk non‐muscle invasive bladder cancer patients receiving BCG and Disitamab Vedotin adjuvant treatments.

## Data Availability

The transcriptome data from de Jong et al. were collected from the study's Supporting Information [4].
