# Supplementary material for: Efficacy of Disitamab Vedotin and Immune Profiles in High‐Risk Non‐Muscle‐Invasive Bladder Cancer With HER2 Overexpression
Source: MedComm (2020). 2025 Oct 7;6(10):e70427. doi: 10.1002/mco2.70427 (PMC12501409; doi:10.1002/mco2.70427)
Supplement: Supplementary file 1 — Supplementary Methods and Materials: Supplementary Table 1: Clinicopathological characteristics in high‐risk non‐muscle invasive bladder cancer patients receiving BCG and Disitamab Vedotin adjuvant treatments. [file MCO2-6-e70427-s001.docx]

**Supplementary Materials for**

**Efficacy of disitamab vedotin and immune profiles in high-risk non-muscle-invasive bladder cancer with HER2 overexpression**

Haoyang Liu^1,#^, Junru Chen^1,#^, Qiyu Zhu^1,#^, Haolin Liu^1^, Yeechun Chuang^1^, Mengni Zhang^2^, Guangxi Sun^1,*^, Hao Zeng^1,*^

^1^Department of Urology, Institute of Urology, Sichuan Clinical Research Center for kidney and urologic diseases, West China Hospital, Sichuan University, 610041, Chengdu, China

^2^Department of Pathology, West China Hospital, Sichuan University, 610041, Chengdu, China

^#^ Haoyang Liu, Junru Chen and Qiyu Zhu contributed equally to this work.

* Correspondence

Guangxi Sun ([sungx077@126.com](mailto:sungx077@126.com)) and Hao Zeng ([kucaizeng@163.com](mailto:kucaizeng@163.com))

**The file includes:**

**Methods**

**References**

**Supplementary Table 1-** Clinicopathological characteristics in high-risk non-muscle invasive bladder cancer patients receiving BCG and Disitamab Vedotin adjuvant treatments.

**Materials and Methods**

**Study design and participants**

This retrospective study included 28 patients with HER2-overexpressed (immunohistochemistry [IHC]≥2+) HR-NMIBC. HER2 overexpression was assessed using IHC staining, with the following scoring criteria: a score of 0 was assigned when no staining or <10% of invasive tumor cells showed incomplete and faint membrane staining. A score of 1+ was given when ≥10% of tumor cells exhibited incomplete and faint membrane staining. A score of 2+ was assigned when ≥10% of tumor cells showed weak to moderate intensity complete membrane staining, or <10% demonstrated strong complete membrane staining. A score of 3+ was given when ≥10% of invasive tumor cells displayed strong complete membrane staining. Risk stratification adhered to European Association of Urology (EAU) guidelines. Propensity score matching was conducted to balance baseline characteristics between DV and BCG-treated groups and enhance the credibility of the results. A matching ratio of 1:1.5 was applied for the DV and BCG cohorts. The parameters included for PSM were as follows: age at diagnosis, gender, height, weight, secondary TURBT, CDK20, CDK5/6, histology variant, HER2 IHC score, tumor size, pT stage, grade, and EAU risk group **(Supplementary Table 1)**.

**Treatment and Assessments**

The BCG instillation schedule consisted of an induction therapy with once-weekly instillations for 6 weeks, followed by once every two weeks for three additional instillations and monthly instillations for 10 months, resulting in a total of 19 instillations within one year. For the subsequent 2 years, the maintenance therapy schedule was set as once every 1-3 month. In this study, the median treatment cycles were 19 and the range was 6-27 times. The BCG stain used was D2PB302.

Baseline clinicopathologic characteristics and outcomes were collected. The primary endpoints were recurrence-free survival (RFS) and twelve-month RFS rate. RFS was defined as the time from treatment to disease recurrence, progression, or death from any cause. The last follow-up date was July 22, 2024. The STROBE reporting guideline was followed. Survival analyses were performed using the Kaplan‒Meier method and compared using the log-rank test.

**transcriptome sequencing data and analysis**

To compare the molecular characteristics of HR-NMIBC tumors with high versus low *ERBB2* mRNA or HER2 protein expression, we analyzed transcriptomic data from 132 primary tumors of BCG-naive HR-NMIBC patients reported by de Jong FC et al.^1^. In the de Jong dataset, high and low *ERBB2* mRNA expression groups were defined using the top and bottom 30% of transcript levels.

Gene set enrichment analysis (GSEA) was conducted to explore gene-set level insights. Immune microenvironment quantification was performed using ESTIMATE and xCell algorithms. Associations among categorical variables were accessed using Fisher’s exact test. All statistical analyses were conducted using R software (Version 4.2.3), with a significance threshold of P < 0.05

**Reference**

1. de Jong FC, Laajala TD, Hoedemaeker RF, et al. Non-muscle-invasive bladder cancer molecular subtypes predict differential response to intravesical Bacillus Calmette-Guérin. *Sci Transl Med*. 2023;15(697):eabn4118.

**Supplementary Table 1. Clinicopathological characteristics in high-risk non-muscle invasive bladder cancer patients receiving BCG and Disitamab Vedotin adjuvant treatments.**

|  |  | **Overall** | **BCG** | **Disitamab Vedotin** | **p-value** |
| --- | --- | --- | --- | --- | --- |
|  |  | **N=28** | **N=17** | **N=11** |  |
| **Age at diagnosis (median [IQR])** |  | 63.500 [51.50, 72.25] | 63.000 [50.00, 72.00] | 66.000 [52.50, 71.00] | 0.638 |
| **Gender (n, %)** | Female | 6 (21.43) | 3 (17.65) | 3 (27.27) | 0.893 |
|  | Male | 22 (78.57) | 14 (82.35) | 8 (72.73) |  |
| **Height (median [IQR])** |  | 165.00 [160.00, 168.00] | 165.00 [160.50, 168.00] | 165.00 [159.00, 166.00] | 0.564 |
| **Weight (median [IQR])** |  | 65.00 [60.00, 70.00] | 65.00 [60.00, 69.750] | 65.00 [61.50, 71.00] | 0.701 |
| **Secondary TURBT (n, %)** | None | 7 (25.00) | 5 (29.41) | 2 (18.18) | 0.823 |
|  | Yes | 21 (75.00) | 12 (70.59) | 9 (81.82) |  |
| **CK20 (n, %)** | Positive | 26 (100.00) | 15 (100.00) | 11 (100.00) | NA |
| **CK5/6 (n, %)** | Negative | 18 (69.23) | 11 (73.33) | 7 (63.64) | 0.921 |
|  | Positive | 8 (30.77) | 4 (26.67) | 4 (36.36) |  |
| **CK20 and CK5/6 (n, %)** | CK20 (+), CK5/6 (-) | 18 (69.23) | 11 (73.33) | 7 (63.64) | 0.921 |
|  | CK20 (+), CK5/6 (+) | 8 (30.77) | 4 (26.67) | 4 (36.36) |  |
| **Histology Variant (n, %)** | Divergent differentiation | 2 (7.14) | 1 (5.88) | 1 (9.09) | 1 |
|  | Pure UC | 26 (92.86) | 16 (94.12) | 10 (90.91) |  |
| **HER2 IHC score (n, %)** | 2+ | 27 (96.43) | 16 (94.12) | 11 (100.00) | 1 |
|  | 3+ | 1 (3.57) | 1 (5.88) | 0 (0.00) |  |
| **Tumor size (mean [SD])** |  | 2.500 (1.34) | 2.625 (1.58) | 2.364 (1.06) | 0.650 |
| **pT stage (n, %)** | Ta | 3 (10.71) | 0 (0.00) | 3 (27.27) | 0.060 |
|  | T1 | 24 (85.71) | 16 (94.12) | 8 (72.73) |  |
|  | Tis | 1 (3.57) | 1 (5.88) | 0 (0.00) |  |
| **Grade (n, %)** | High | 25 (92.59) | 14 (87.50) | 11 (100.00) | 0.638 |
|  | Low | 2 (7.41) | 2 (12.50) | 0 (0.00) |  |
| **EAU risk group, (n, %)** | High risk | 27 (100.00) | 17 (100.00) | 11 (100.00) | NA |

BCG, Bacille Calmette-Guerin; IQR, interquartile range; n, number; TURBT, transurethral resection of bladder tumor; CK, cytokeratin; IHC, immunohistochemistry; UC, urothelial carcinoma; SD, standard deviation; Tis, Tumor in situ; EAU, European Association of Urology; NA, not available.
